# Early arriving males wait longer for a mate than later arrivals: the case of a migratory monogamous passerine bird species

**DOI:** 10.1007/s10164-017-0531-y

**Published:** 2017-11-14

**Authors:** Cezary Mitrus

**Affiliations:** Department of Zoology, University of Rzeszów, Zelwerowicza 4, 35-601 Rzeszów, Poland

**Keywords:** Arrival time, Protandry, Age of male, Mating success, Red-breasted Flycatcher* Ficedula parva*

## Abstract

Individuals that arrive earlier on the breeding grounds may obtain many advantages but they also have to spend time waiting for a mate. I studied the waiting times of male red-breasted flycatchers *Ficedula parva*, a small, migratory, sexually dichromatic passerine bird species under natural conditions (Białowieża National Park, Poland) in relation to year, arrival time, age of male and morphological parameters. The length of waiting time was dependent on males’ arrival time. The males which arrived later waited a shorter time for females than earlier arrivals. In some years older males spent more time waiting for mates than younger males, but in other years they waited for shorter times. A significant interaction between age of male and year was also observed. Despite the significantly earlier arrival of older males, the waiting time was not related to male age. The waiting time was also not related to body biometric parameters of the male. Despite waiting longer, early male red-breasted flycatchers have an advantage over later arrivals given this greater chance of mating.

## Introduction

The earlier arrival of males at breeding areas relative to females (protandry) is a common phenomenon observed in many migrating bird species (Morbey and Ydenberg 2001; Gunnarsson et al. 2004; Kokko et al. 2006; Newton 2008; Harnos et al. 2015). Several hypotheses have been proposed to explain protandry in birds relating to greater mating opportunity (Wiklund and Fagerstrom 1977), rank advantage (Ketterson and Nolan 1976; Kokko 1999) and the susceptibility hypothesis (Møller 2004). Individuals that arrive earlier may obtain many advantages, such as higher quality territories (Potti and Montalvo 1991; Aebischer et al. 1996; Smith and Moore 2005) which can increase the probability of mating success (Møller 1994; Lozano et al. 1996; Kokko 1999) and breeding success (Perrins 1970; Slagsvold and Lifjeld 1988). Early arrivals, however, can also pay some costs because severe weather early in the season can often lead to higher rates of mortality (Møller 1994) and they also have to wait for females to arrive.

So far, most studies related to protandry have concentrated on the advantages with less attention being paid to the time that males wait for females and the factors related to this (Møller 1994; Kokko 1999; Morbey and Ydenberg 2001; Møller 2004). After arrival, while they wait for mates, males spend energy occupying, advertising and defending their territory (Morbey and Ydenberg 2001). Many studies have concentrated on factors influencing male arrival time at breeding territories (Møller 1994; Kokko 1999; Mitrus 2007, Pulido 2007) and how this relates to mating success (Aebischer et al. 1996; Lozano et al. 1996; Kokko 1999), but there is a lack of studies relating to the time that males spend from arrival to mating. The mating success of males can be related to many factors, e.g. male age and characteristics, and quality of the territory. It is interesting to consider what factors determine how long males wait for mates: is it age, arrival time or phenotypic traits of individuals? Such questions can addressed in long-distance migratory bird species like the red-breasted flycatcher *Ficedula parva*, where males arrive at the breeding areas before the females. *F. parva* is a migratory, small, sexually dichromatic passerine bird species, wintering in the Indian sub-continent, and males arrive at the breeding areas before females (Cramp and Perrins 1993; Mitrus et al. 2005). The earliest males arrive in eastern Poland in late April to early May (Mitrus et al. 2005), and within these, older males arrive significantly earlier (Mitrus 2007). After arrival, males sing and defend territories while waiting for the opportunity to mate. In the Białowieża National Park, red-breasted flycatchers breed solitarily in natural tree cavities (Mitrus and Soćko 2004) in deciduous or mixed stands, in low densities of up to 2.0 pairs/10 ha (Wesołowski et al. 2010, 2015).

I analysed variation in male waiting time in the red-breasted flycatcher under natural conditions to address the following questions: Is male waiting time related to arrival time and depend on the age of the male? Is waiting time connected with biometric characteristics of males? And finally a more general question: is it advantageous for males to arrive early and wait for a mate?

## Study area

Observations were carried out from 2000 to 2010 in Białowieża Forest (NE Poland — 52°41′N, 23°52′E), in three study plots (total area 795 ha). The plots were characterised by old-growth lime-oak-hornbeam *Tilio*-*Querco*-*Carpinetum* stands with many standing and fallen dead trees (Tomiałojć 1991) and comprised mainly hornbeam *Carpinus betulus*, small-leaved lime *Tilia cordata*, pendunculate oak *Quercus robur,* Norway maple *Acer platanoides* and Norway spruce *Picea abies* (Wesołowski et al. 2010).

## Materials and methods

From the end of April to the third decade of May, daily, on the study plots, one to four persons searched for newly arriving males by listening for the songs of individual red-breasted flycatchers. The place and time were noted and territories were considered occupied if males were observed for 3 or more days. In the statistical analyses, we standardised arrival time in each year by using the arrival date of the first male to arrive as day 1. The active territories with males were checked every day to determine when mating occurred. Mated males of the red-breasted flycatcher ceased singing (Mitrus et al. 2012), therefore, if a male was seen to have stopped singing and was observed with female, or seen copulating, then it was designated as mated. The difference between the date of mating and the arrival time was defined as the waiting time for a mate.

Males were captured as soon as possible after arrival using a concealed tape-recorder broadcasting conspecific song to lure birds into a mist net (16-mm mesh, dimensions 6 × 25 m) within a male’s territory. The birds were marked with a unique combination of one aluminium and three coloured plastic rings. The following measurements were taken: wing length (maximum wing chord in mm, Kelm 1970); body mass, with a 30-g Pesola spring balance (to the nearest 0.25 g) and tarsus length with sliding callipers (to the nearest 0.1 mm). All birds were aged based on plumage coloration: older males (after the 2nd year) exhibit an orange patch of feathers extending from the throat to the breast, and bluish feathers under the eye and on the neck. Young males (2nd year) exhibit female-like plumage coloration: the throat and breast lacks orange plumage and there is no blue plumage on the face (Svensson 1992). In the population studied, older males were more common than younger males (3:1) (Mitrus et al. 2006). Most ringed young and older males did not come back to the study area the next year, and most adults were new individuals that came from other places. Therefore, in many cases one individual was observed for only 1 year, and there were extremely few repeatedly measured individuals. The data related to the one individual was use in the statistical analyses only once.

To determine factors affecting the waiting times of males, two generalized linear models (GLM) with Poisson distributions and log link functions were constructed: first, where year, age and time of arrival were used as independent factors, and a second to determine the influence of biometric traits of males (body mass, wing and tarsus length) on the waiting time. Two separate models were used because of lack of biometric data for some young males in 2 years (2005 and 2008). To determine differences in mating success in relation to time of arrival, males were divided into two groups (early and late arrivals) based on median and quartiles (lower and higher) of arrival time. All statistical analyses were undertaken using Statistica for Windows v.10.0.

## Results

**Fig. 1:**
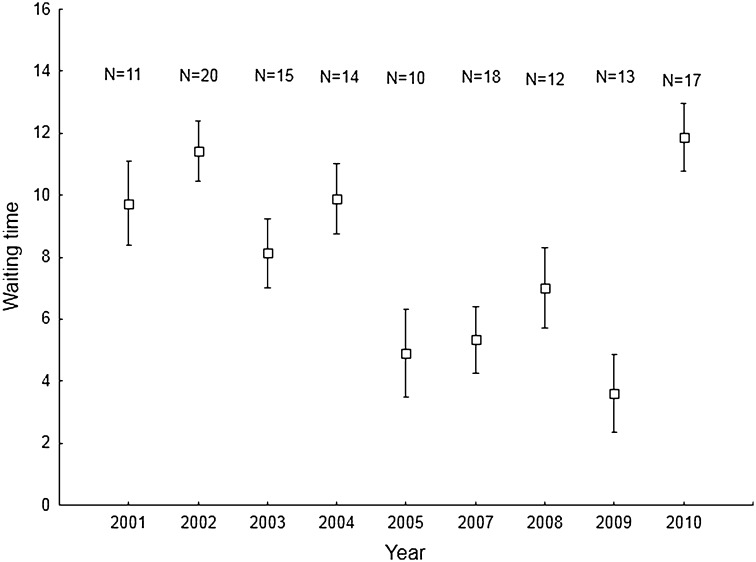
Changes of waiting time (mean ± SE) in subsequent years

**Fig. 2:**
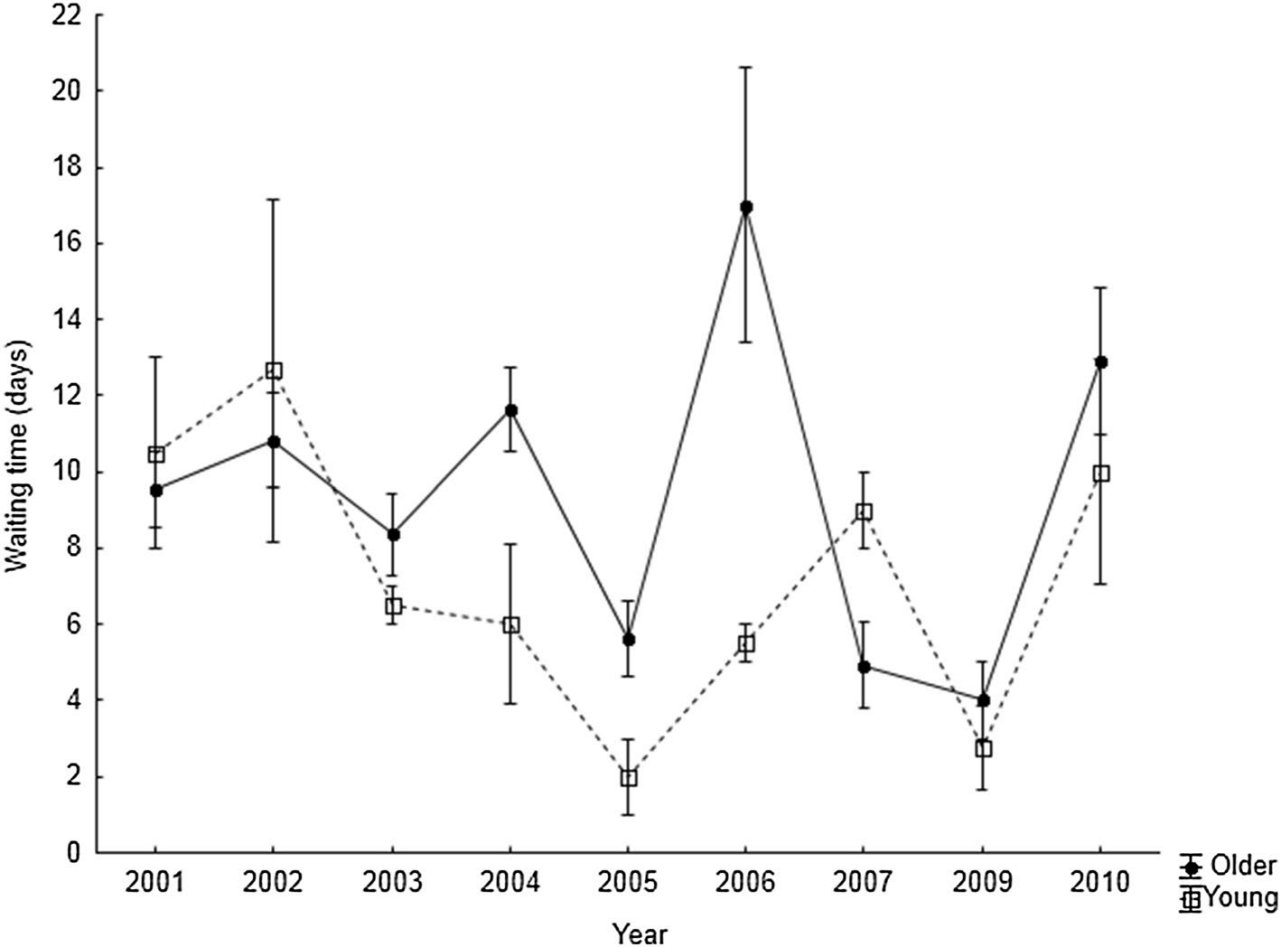
Changes in mean waiting times in relation to the age of the male and breeding season

I determined the arrival time and waiting time for 150 mated males (119 older and 31 young). The waiting time varied from 0 to 24 days (mean = 8.5 ± 5.4), 71% of males waited for a mate for up to 10 days. Length of waiting time was dependent on year and the time of arrival of the male (Table 1). In consecutive years, male waiting time can differ twofold, for example in the years 2004 and 2005, and 2009 and 2010 (Fig. 1). A significant interaction between the age of the male and year was observed (Table 1) and in some years older males spent a shorter time waiting for mates than young males; in other years it was longer (Fig. 2).

**Table 1:** Results of generalized linear model testing the relationships between arrival time, year, male age and sex with the waiting time for a mate

Effect	Degrees of freedom	Wald statistic	*p*
Intercept	1	368.9	< 0.001
Arrival time	1	41.9	< 0.001
Year	8	25.2	0.001
Age	1	0.13	0.71
Year*Age	8	21.26	0.006

**Fig. 3:**
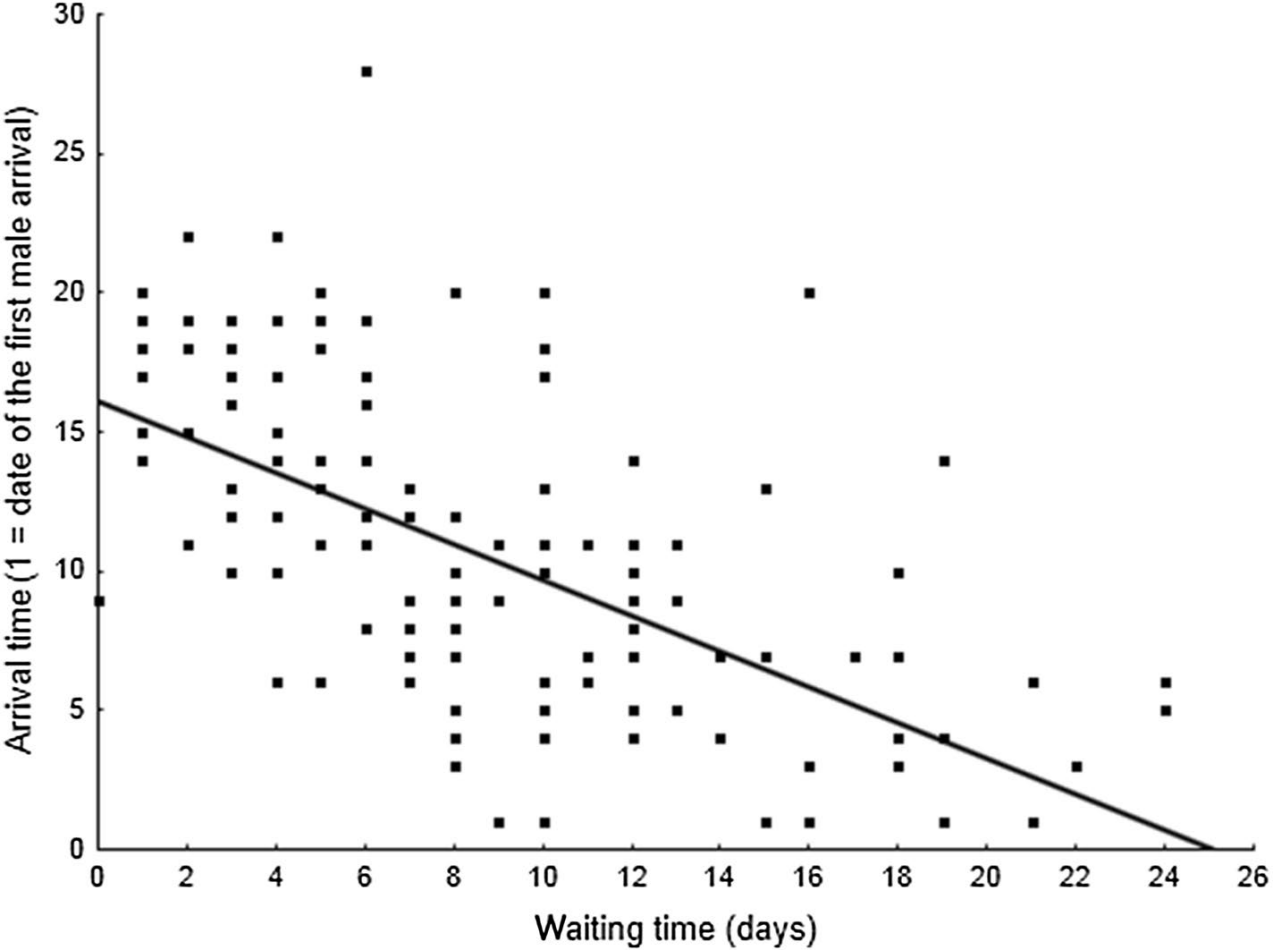
Relationship between male arrival time and waiting time for a mate. Data for young and older males combined

The males which arrived later waited a shorter time for females than earlier arrivals (*r* = − 0.60, *N* = 150, *p* < 0.001, Fig. 3). Despite the significantly earlier arrival of older males (*t* = − 5.29, *df* = 148, *p* < 0.001) the waiting time was not related to male age. The waiting time was also not related to body mass, wing or tarsus length of the male (Table 2). Early arrivals (before day 5 of arrival time, lower quartile) were more often mated (80.0 vs 34.3%, *χ* ^2^ with Yates’s correction = 14.83, *N* = 315, *p* < 0.01) than males which arrived late (after 13 days of arrival time, higher quartile).

**Table 2:** Results of generalized linear model testing the influence of biometric parameters of males on the waiting time for a mate

Effect	Degrees of freedom	Wald statistic	*p*
Intercept	1	1.27	0.26
Wing length	1	2.99	0.08
Tarsus length	1	1.58	0.21
Body mass	1	1.88	0.17

## Discussion

I have documented in this paper that early arriving males of the red-breasted flycatcher have to wait longer for a mate than later arrivals. Firstly, the main reason for this is that early males arrive before females (protandry) and thus the waiting time is partly dependent on female arrival time. Therefore, it is also obvious that in the case of early arrivals, because of the later arrival of females, a longer time between arrival and mating was observed. In the red-breasted flycatcher the later arrival of females at the breeding area is also supported by evidence from spring migration records. A recent study from a stopover site in northern Turkey showed that females migrated on average 6 days later than old males (Erciyas-Yavuz et al. 2015). Studies on other species indicate that arrival times of males can be mostly determined by differences in wintering areas and the timing of the start of spring migration rather than by differences in the speed of migration (Coppack and Pulido 2009). Despite this, Maggini and Bairlein (2012) proved that in northern wheatears *Oenanthe oenanthe*, protandry has an endogenous basis with an innate earlier spring departure of males than females.

The waiting time in the red-breasted flycatcher also varied in relation to the year. This is supported by an earlier study where arrival time of first males at the breeding area differed by as much as 9 days between consecutive years (Mitrus et al. 2005). The date of arrival and numbers arriving was mostly influenced by weather conditions in the wintering areas and over the migration route, which can vary significantly between years (Mitrus et al. 2005). The arrival time of males also determines the date of the first egg laying in the season (Mitrus 2006). It is indicated that in the years when males and females arrived later they had a shorter time to start reproduction, and the gap between arrival and mating time is shorter. An interaction of male age and year on waiting time was observed. This is related to the variation and differences between arrival time of old and young males in different years (Mitrus 2007). In some years young males arrived on average less than 3 days later, and in other years more than 9 days (Mitrus 2006, 2007): this fact reflects changes and variation in waiting time.

The time of arrival of migrating bird species at their breeding areas is often considered and in many studies most attention is paid to the advantages rather than the costs of being first (Potti and Montalvo 1991; Møller 1994; Aebischer et al. 1996; Lozano et al. 1996; Johnson 1997; Kokko 1999; Smith and Moore 2005). In some studies, the cost of early arrival is connected with severe conditions in early spring, i.e. less food available, worse weather conditions (Møller 1994). Rather rarely within these costs was the waiting time for a partner mentioned. While waiting for a mating opportunity, males have to advertise themselves by singing and defend their territory against intruders. However, studies of a closely related species, the pied flycatcher *Ficedula hypoleuca*, indicate that song is not energetically costly in comparison to the daily energy budget (Ward et al. 2004). Yet it should be remembered that during singing the male cannot also forage and thus indirectly this activity is costly.

The time of arrival of the red-breasted flycatcher males influenced mating success, and early arrivals had a greater chance of obtaining a mate than males arriving later. The time of arrival can be determined by various factors. At the individual level, arrival time was most strongly related to male age (Mitrus 2007), a finding also confirmed in this work. In earlier studies, no differences were found in characteristics of territories in relation to the arrival time of males (Mitrus et al. 2006). However, early arrivals in this species also start breeding earlier (Mitrus 2006). Under natural conditions in the Białowieża Forest, the most important factor influencing breeding success is predation (Walankiewicz 2002; Wesołowski 2002; Mitrus and Soćko 2008; Wesołowski and Tomiałojć 2005). In the case of the red-breasted flycatcher, most losses were noted during egg laying and incubation (Mitrus and Soćko 2008); thus, early arrivals and breeders have a greater chance of making a repeat breeding effort in the same season, and hence a greater chance of achieving breeding success.

Protandry seems to be under sexual selection pressure and is observed in relation to sexual size and plumage dimorphism, and polygyny (Kissner et al. 2003; Rubolini et al. 2004). In the red-breasted flycatcher, sexual dimorphism in both size and plumage is observed (Svensson 1992) and, although it is considered to be a socially monogamous species (Mitrus and Soćko 2005), in more than 24% of clutches extra-pair nestlings were found (Mitrus et al. 2014). More extra-pair nestling were found in the broods of young males which bred significantly later (Mitrus et al. 2014). Moreover, older, earlier arriving males were always found to be the extra-pair partner in broods with extra-pair young, irrespective of the age of the social father (Mitrus et al. 2014). Similarly in the pied flycatcher, males breeding early were often socially polygynous and were attaining extra-pair paternity and so increased their reproductive success (Canal et al. 2012). Thus, female sexual choice may solidify selection on older males which arrive earlier. This is also supported by observations that the occurrence of extra-pair paternity is associated with the degree of protandry in other passerine species (Coppack et al. 2006). Thus, protandry may have evolved from selection on the relative arrival timing of males and females.

In conclusion, it seems that in the case of the red-breasted flycatcher, it is more advantageous for males to arrive earlier, spend more energy on territory defence, singing and waiting for a longer time before mating, than to arrive later and to have less chance of mating and breeding success.
